# *In silico* pharmacological study of AQP2 inhibition by steroids contextualized to Ménière’s disease treatments

**DOI:** 10.3389/fneur.2023.1270092

**Published:** 2023-10-19

**Authors:** Robin Mom, Stéphane Réty, Vincent Mocquet, Daniel Auguin

**Affiliations:** ^1^Laboratoire de Biologie et Modélisation de la Cellule, École Normale Supérieure de Lyon, CNRS, UMR 5239, INSERM U1293, Université Claude Bernard Lyon 1, Lyon, France; ^2^Research Group on Vestibular Pathophysiology, CNRS, Unit GDR2074, Marseille, France; ^3^Laboratoire de Physiologie, Ecologie et Environnement (P2E), UPRES EA 1207/USC INRAE-1328, UFR Sciences et Techniques, Université d’Orléans, Orléans, France

**Keywords:** steroids, aquaporin, Ménière disease, vitamin D3, oestradiol

## Abstract

Ménière’s disease (MD) is characterized by an abnormal dilatation of the endolymphatic compartment called endolymphatic hydrops and is associated with fluctuating hearing losses and vertigo. Corticosteroid treatment is typically administered for its anti-inflammatory effects to MD patients. However, we recently described for the first time a direct interaction of two corticosteroids (dexamethasone and cortisol) with human AQP2 which strongly inhibited water fluxes. From these initial studies, we proposed an AQPs Corticosteroids Binding Site (ACBS). In the present work, we tested the interaction of 10 molecules associated to the steroid family for this putative ACBS. We observed a wide diversity of affinity and inhibitory potential of these molecules toward AQP2 and discussed the implications for inner ear physiology. Among the tested compounds, cholecalciferol, calcitriol and oestradiol were the most efficient AQP2 water permeability inhibitors.

## 1. Introduction

Endolymphatic hydrops (EH) is an abnormal dilatation of the endolymph compartment, observed on magnetic resonance imaging (MRI) of a very large majority of Ménière’s Disease (MD) patients [EH in 84% of MD ears (1)] and other hearing disorders such as sensorineural hearing losses (SNHL) [EH in 90% of low tone SNHL ears (2) and in 68% of idiopathic sudden SNHL ears (3)]. This phenomenon is considered as a marker of MD (4, 5), even if its direct contribution to the symptoms of the disease remains to be proven (6, 7). Altogether, these data highlight a strong correlation between rupture of water fluxes homeostasis in the inner ear and hearing disorders.

The inducing factors of EH are still unknown. However, it is thought to result from a massive influx of water from perilymph to endolymph (8, 9). Different causes have been put forward to explain this phenomenon, without any being particularly prioritized. One hypothesis involves the modulation of water fluxes between lymphatic compartments through the alteration of membrane water permeability. This permeability directly relies on the regulation of the transmembrane water channel family of aquaporins (AQPs) (10, 11). Among them, AQP2 is very interesting due to its role in the control of transmembrane water fluxes (12–14) but also because it is expressed in areas of fluid exchange between perilymph and endolymph (9, 13, 15–18).

Different pharmacological approaches have been developed in an attempt to absorb the accumulation of water responsible for EH. The administration of diuretics is part of the MD patient care recommendations (19) to reduce hydrops and alleviate the associated vertigo syndrome. Trans-tympanic administration of corticosteroids such as dexamethasone (DXM) is also one of the therapeutic approaches recommended in MD, when conventional antivertigo treatments have failed (19). This approach has shown significant benefits in the treatment of MD (20–23). The main mechanism of action put forward to explain the effect of corticosteroids is their anti-inflammatory effect; however for some patients no relief could be obtained which questioned their efficiency in MD treatment (22, 24–27).

In previous studies, we highlighted a functional modulation of AQP2 water channeling by the direct interaction of its extra-cellular surface with synthetic (DXM) (28) and naturally produced (cortisol) (29) corticosteroids through *in silico* simulation approaches. These interactions occurred at physiological concentrations (mean *K*_D_ of 317.7 nM and of 239.17 nM for dexamethasone and cortisol respectively) and induced a significant reduction of the channel water permeability. Corticosteroids were already known for their regulatory action on some AQPs transcripts level (30, 31). However, with these studies we bring strong evidence that corticosteroids can also alter AQP2 function in directly impeding water fluxes through this channel which has never been shown so far, to our best knowledge. Based on the structural homology existing between mineralocorticoid receptors (MRs), glucocorticoid receptors (GRs), cholesterol consensus motif (CCM), and the extra-cellular vestibules of AQPs, we proposed a putative AQPs Corticosteroids Binding Site (ACBS) (29). In the present study, we screened ten steroids for their affinity for AQP2 ACBS and for their impact upon the water permeability of the channel. We then discussed the nature of the molecular mechanism involved and the relevance of such regulation for the MD community.

## 2. Method

### 2.1. Molecular dynamics simulations

All simulations were performed with GROMACS (v.2018.1) (32) in a CHARMM36m force field (33). The systems were built with CHARMM-GUI interface (34, 35). The first minimization step was followed by six equilibration steps, during which, restraints applied on the protein backbone, side chains, and lipids were progressively removed before the production phase was performed without restraint. Pressure and temperature were kept constant at 1 bar and 310.15 Kelvin, respectively, using the Berendsen method during equilibration and Parrinello–Rahman and Nosé–Hoover methods during production. The Lennard–Jones interaction threshold was set at 12 Angströms (Å) and the long-range electrostatic interactions were calculated through the particle mesh Ewald method.

The tetrameric assembly of AQP2 (pdb: 4nef) was inserted into the POPC bilayer, solvated with TIP3 water molecules, and 150 mM of KCl for the “control” condition. For the 10 other conditions, ab additional 4 steroid molecules were manually placed inside the extra-cellular vestibules of AQP2 following the same procedure as in our previous work (28, 29) hence leading to one steroid per monomer. The steroids tested were: cortisol (COR), solu-medrol (SLM), dexamethasone (DXM), methylprednisolone (MPR), cortisone (CON), androsterone (AND), progesterone (PRG), oestradiol (OES), and vitamin D3 in the inactive form cholecalciferol (VD3I) and the active form calcitriol (VD3A). The 10 systems were then simulated for 200 nanoseconds.

### 2.2. Analysis

#### 2.2.1. Water permeability

To monitor water molecules displacement along the trajectories, the MDAnalysis library was used (36, 37). From these water coordinates are derived water counts and permeability coefficients (*pf*). Permeability coefficients were calculated according to the collective coordinate method (38).

#### 2.2.2. Inhibitory potential coefficient

To estimate the inhibitory potential of the molecules tested, a coefficient that we named the water fluxes Inhibitory potential coefficient (*WIP*) was calculated as follows:


WIP=ΔcountsKDmedianNA∗Vm

with Δ_counts_: the difference between the mean number of water molecules crossing the whole 30 Angströms (Å) long trans-membrane pore section in the control condition and the condition docked with the molecule tested; *K*_Dmedian_: the median *K*_D_ computed over the 200 nanoseconds trajectory of the tested molecule docked to AQP2; *N*_A_: the Avogadro constant and *V*_m_: the water molar volume. The coefficient is expressed in cm^3^ s^−1^ nM^−1^.

#### 2.2.3. Free energy profiles

Water-free energy profiles were extrapolated from the logarithm function of the water counts inside the pore with the *z*-axis as a reaction coordinate (39, 40). The pore is divided along the reaction coordinate (*z*-axis) in slices of 0.5 Angströms (Å). The average density of water molecules in each slice is then computed over the 200 nanoseconds of simulation and the Gibbs free energy *G*(*z*) is obtained as follows:


$$G\left(z\right)=-KT\;\ln\frac{\rho \left(z\right)}{\rho_{\mathrm{bulk}}}$$

where *K*, *ρ*_bulk_, and *T* represent the Boltzmann constant, the bulk density, and the absolute temperature, respectively.

#### 2.2.4. Binding free energy and dissociation constant

The binding affinity of steroids to AQP2 was evaluated directly from the structure, extracted from the 200 nanoseconds molecular dynamics trajectories every nanosecond, with the PRODIGY-LIG program (41). PRODIGY-LIG evaluates the contacts between ligand and protein and computes free binding energy from a reliable empirical equation (41).

Dissociation constant (*K*_D_) values were obtained from the binding free energies as follows:


$$K_{D}=e^{\left(\frac{-\Delta G_{s}}{RT}\right)}$$

with Δ*G*_S_, *R*, and *T* the binding free energy, the perfect gas constant, and the temperature, respectively.

#### 2.2.5. Other properties

Hydrogen bonds were computed with GROMACS tools.

#### 2.2.6. Statistical analysis

All statistical analyses were performed using the R programming language. Bonferroni *post hoc* correction after the Wilcoxon test was used to compare conditions together.

For all the conditions, the 200 nanoseconds trajectories were divided into 10 nanoseconds sub-trajectories, and the analysis was performed for each monomer hence yielding 80 repetitions per condition.

## 3. Results

### 3.1. Comparison of ten steroids for their affinity to the AQPs corticosteroids binding site

**Figure 1:**
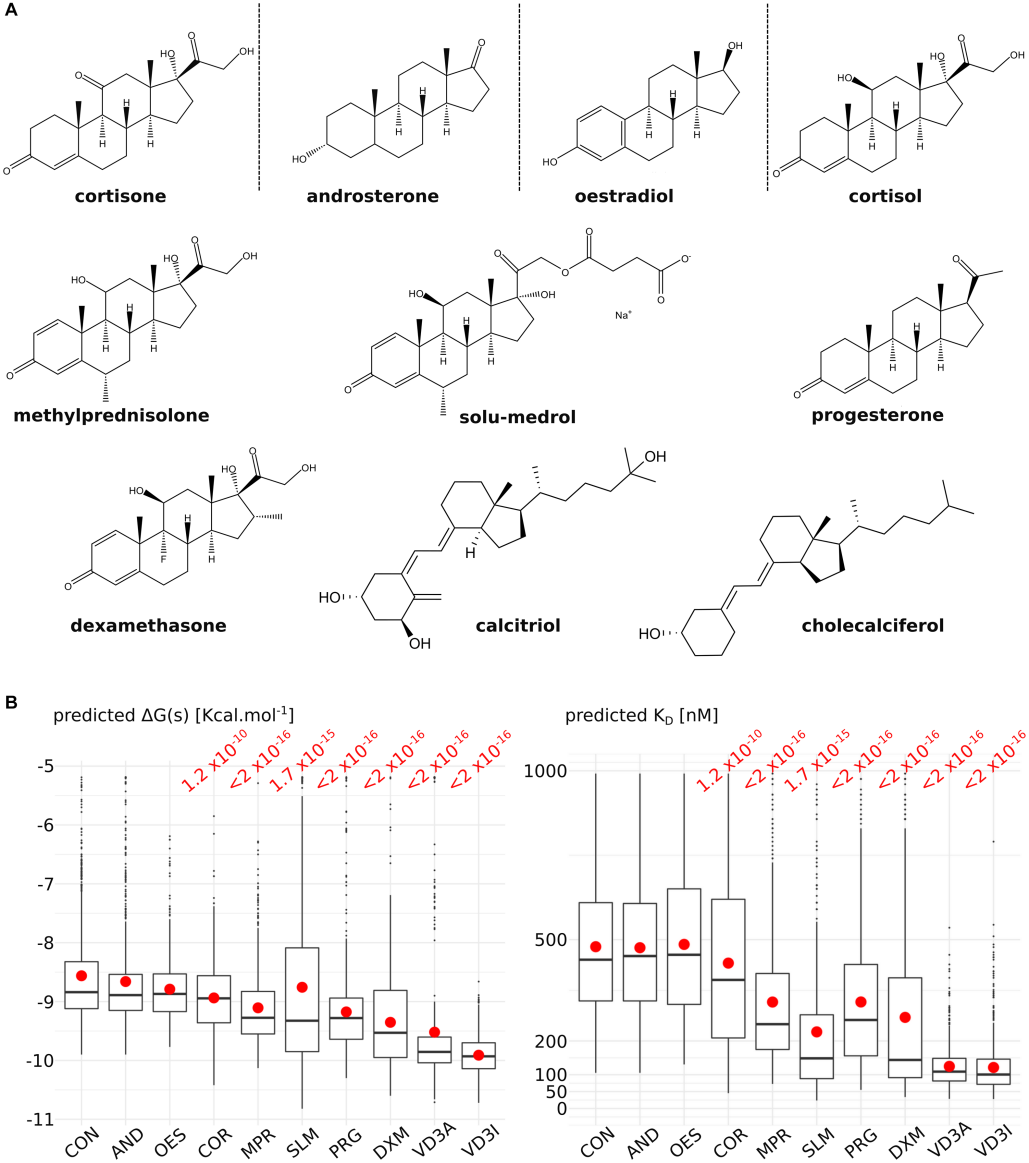
Comparison of nine steroids for their affinity to the AQPs corticosteroids binding site (ACBS). **(A)** Representation of the nine steroids docked into AQP2 ACBS. For each of them, 200 nanoseconds of trajectory were simulated and sampled every nanosecond for binding free energy and *K*_D_ calculation. **(B)** Graphical representation of the binding free energies and *K*_D_ for cortisone (CON), androsterone (AND), oestradiol (OES), cortisol (COR), methylprednisolone (MPR), solu-medrol (SLM), progesterone (PRG), dexamethasone (DXM), active form of vitamin D3 calcitriol (VD3A), and inactive form of vitamin D3 cholecalciferol (VD3I). Means are indicated by a red point and medians by the horizontal bars. All conditions were compared to one another with a non-parametric Wilcoxon test. *p*-values are indicated in red when a significant difference appeared between one condition and the “CON” condition.

To test the relevance of the predicted ACBS, 10 molecules from the steroid family were docked into AQP2 ACBS: 5 corticosteroids (4 glucocorticoids: cortisol, dexamethasone, methylprednisolone and solu-medrol; and 1 mineralocorticoid: cortisone), androsterone, progesterone, oestradiol and vitamin D3 inactive (cholecalciferol) and active (calcitriol) form (Figure 1A). Predicted binding free-energy ranges the interaction between AQP2 extra-cellular surface and the steroid molecule from the least to the most stable as follows: cortisone, androsterone, oestradiol, cortisol, methylprednisolone, solu-medrol, progesterone, dexamethasone, calcitriol, and cholecalciferol. From these binding free energies were calculated dissociation constants (*K*_D_) ranging from 590 nM to 101 nM (median values calculated over the whole 200 nanoseconds simulation sampled every nanoseconds) for cortisone and cholecalciferol, respectively (Figure 1B).

### 3.2. Steroids impact upon AQP2 water permeability

**Figure 2:**
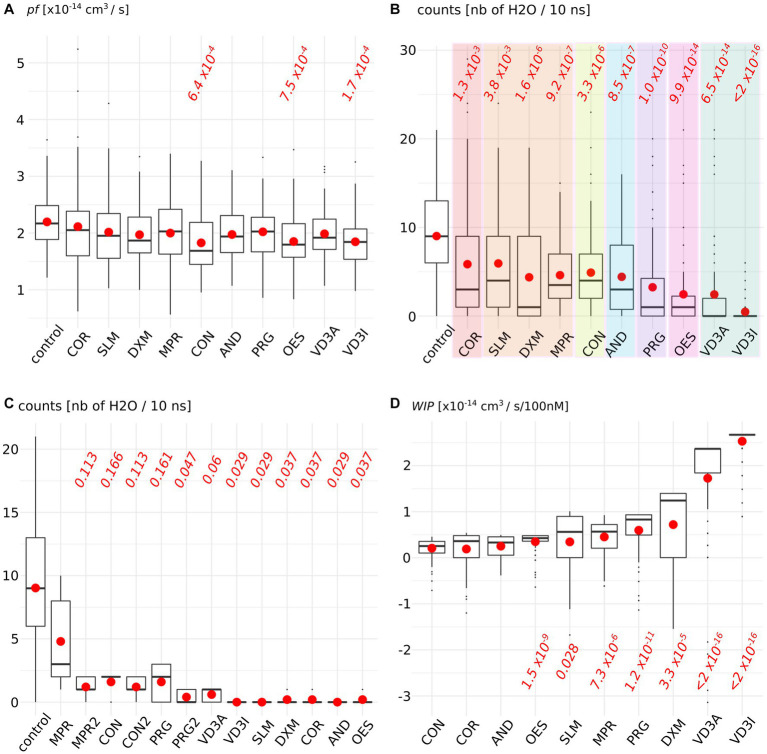
Steroids impact upon AQP2 water permeability. **(A)** Permeability coefficient *pf* is calculated for each condition. **(B)** Number of water molecules crossing the whole 30 angstrom-long trans-membrane conducting pore section. Each family of steroids is colored differently: from left to right: in red, naturally produced glucocorticoid; in orange, synthetic glucocorticoid; in yellow, naturally produced mineralocorticoid; in blue androgen; in purple, progestogen; in pink, estrogen, and in green vitamin D3. **(C)** Number of water crossing the whole transmembrane section for all the simulation time in the control condition or when the steroid is forming hydrogen bonds with arginine 187 of AQP2 ACBS. **(D)** Water fluxes inhibitory potential coefficient of the tested molecules. The estimated *K*_D_ is integrated into the water permeability reduction (see methods) in order to give the estimated water fluxes inhibition for an arbitrarily chosen 100 nM concentration of the steroid tested. For both indicators, all conditions were compared to one another with non-parametric Wilcoxon test. *p*-values are indicated in red when a significant difference appeared between one condition and the “control” [or with the “CON” condition for *WIPs* in **(D)**].

Figure 2 depicteds the impact of the AQP2–steroid interaction upon two water permeability indicators. The permeability coefficient *pf* is traditionally used to estimate the water permeability of AQPs and is derived from the diffusion of water inside the conducting pore (38) (Figure 2A). From this first approach, three molecules significantly reduced water fluxes compared to the control condition: cortisone, oestradiol, and cholecalciferol. To complement this indicator, we also counted the number of water molecules crossing the whole 30 angstroms-long trans-membrane pore section (Figure 2B). This more straightforward methodology revealed significant impacts of all the tested molecules upon water fluxes compared to the control condition but within different orders of magnitude: a first group is constituted by cortisol and solu-medrol and is associated with *p* values around 1 × 10^−3^; then come dexamethasone, methylprednisolone, cortisone, and androsterone with *p* values close to 1 × 10^−6^; progesterone follows with a *p* value of 1.0 × 10^−10^; then oestradiol and calcitriol are associated with a *p* value around 1 × 10^−14^; and finally, cholecalciferol reduces water fluxes the most significantly as indicated by the *p* value <2.0 × 10^−16^. These water permeability coefficients were calculated over the whole trajectories, hence integrated ACBS-bound and ACBS-unbound states. To evaluate the efficiency of the inhibition of each molecule when bound to the ACBS, an ACBS-bound state was defined by the establishment of hydrogen bonds between the steroid and one of the most conserved residues of the ACBS: the arginine 187. This arginine is located in the most stringent part of the conducting pore in the extra-cellular half of the AQP. It corresponds to high protein–water interactions and is called the aromatic/arginine (ar/R) constriction (40). This constriction is typically composed of one arginine (arginine 187 in AQP2) and at least one aromatic residue (histidine 172 in AQP2). This arginine is one of the most conserved residues in aquaporins and of the ACBS (29, 42), and constitutes a key component of the AQP selectivity (43). Hence, we compared the number of water molecules crossing the whole trans-membrane section of the channel of 50 nanoseconds long portions of trajectory during which the tested molecule establishes the highest frequency of hydrogen bonds with the arginine of the ar/R constriction (Figure 2C). We can observe that all steroids did not inhibit water fluxes with the same efficiency when bound to the ACBS. Interestingly, three of the tested molecules did not display the highest inhibition of water fluxes when forming hydrogen bonds with this arginine (Figure 2C conditions MPR, CON, and PRG) but rather with residues of extra-cellular loop A and C (Figure 2C conditions MPR2, CON2, and PRG2). To better evaluate the impact of each molecule upon AQP2 water permeability, water count differences with the “control” condition were pondered by the corresponding median *K*_D_ hence giving the estimated AQP2 water fluxes reduction as a function of the steroid concentration (see methods, Figure 2D). For an arbitrary concentration of 100 nM of the tested molecule in solution, the predicted AQP2 water fluxes inhibition coefficients (Water fluxes Inhibitory Potential coefficient: *WIP*) clearly indicate both calcitriol and cholecalciferol as the most significantly relevant putative inhibitors of AQP2 (Figure 2D).

### 3.3. Molecular mechanism of AQP2 water permeability reduction by vitamin D3

**Figure 3:**
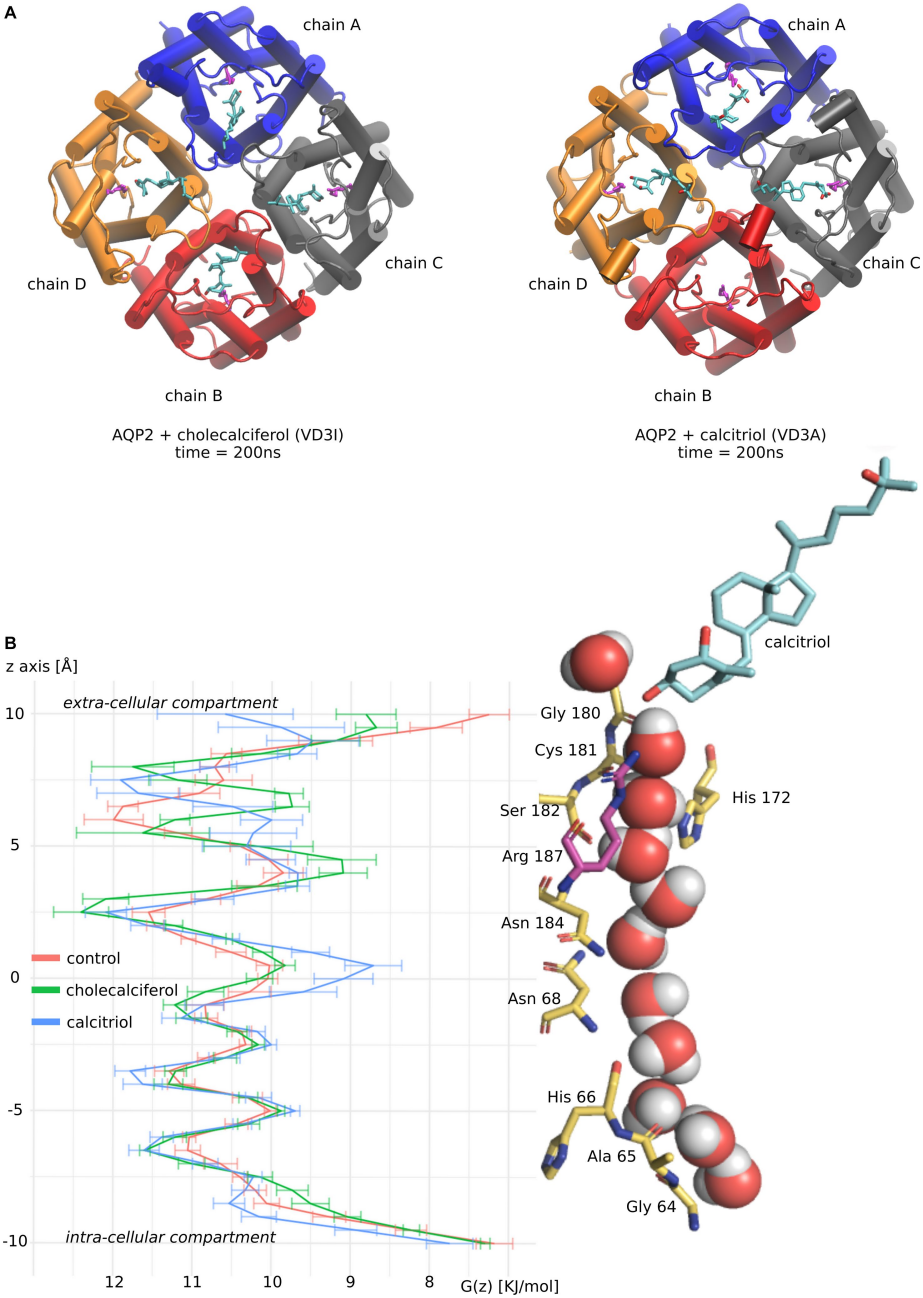
Molecular mechanism of AQP2 water permeability reduction by vitamin D3. **(A)** Schematic representation of vitamin D3 inactive form (cholecalciferol) and active form (calcitriol) in interaction with AQP2 extra-cellular vestibule. Vitamin D3 is colored in cyan. Each AQP2 monomer is represented in a different color. The arginine of the ar/R constriction in AQP2 is colored magenta. The two representations correspond to snapshots of the end of the molecular dynamics simulations at time *t* = 200 nanoseconds. **(B)** The channel water-free energy profiles for “control,” “cholecalciferol,” and “calcitriol” conditions were calculated over 50 nanoseconds parts of the trajectories. These 50 nanoseconds parts of trajectories correspond to simulation portions where the frequency of ar/R arginine 187 – vitamin D3 hydrogen bonding is maximized. For “control” where no steroids were docked the first 50 nanoseconds were used. On the right side of the figure, a schematic representation of AQP2 pore-lining residues in interaction with water molecules and calcitriol (colored in cyan) are aligned with the water free-energy profiles. Arginine 187 of the ar/R constriction is colored in magenta. The representation was made from a snapshot of the trajectory portion used for water-free energy profile calculation at time *t* = 200 nanoseconds.

As both active and inactive forms of vitamin D3 stand out from the other molecules tested by both their affinity for AQP2 ACBS and their inhibition of water fluxes, we focused our analysis on these two molecules (Figure 3).

To better understand molecular mechanisms underlying such modulation of AQP2 water permeability by vitamin D3, we compared the water free-energy profiles of the conditions corresponding to four cholecalciferol or four calcitriol molecules docked in AQP2 ACBS (Figure 3A) with the “control” condition (without steroids docked into AQP2) (Figure 3).

Contrary to cholecalciferol which stayed in an ACBS-bound state in each of the four sub-units of AQP2 during the whole simulation, one molecule of calcitriol left the ACBS. This is illustrated by the schematic representation of the AQP2 tetramer and the steroids at the end of the simulations (Figure 3A). This difference could be explained by the more hydrophobic nature of cholecalciferol, better accommodated by the AQP2 ACBS. The higher stability conferred could be accountable for the difference in *WIP* between the two molecules, even though calcitriol still inhibits AQP2 water permeability very significantly.

The water profiles show the characteristic alternation of water interaction sites constituted by backbone carbonyl oxygens or polar side chains (for instance, the central asparagines of the NPA – asparagine, proline, alanine – motifs are well known for their interaction with water molecules (44) and clearly correspond to local minima on free energy profiles) with energetic barriers corresponding to hydrophobic parts of the channel. The most stringent part of the conducting pore corresponds to the aromatic/arginine (ar/R) constriction (40). In previous work, we showed that cortisol was reducing water fluxes mainly through the increase of hydrophobicity of the channel rather than by steric constraints (29). This effect was located at the interaction site but also spread throughout the whole conducting pore because of the attenuation of the positive charge of the arginine of the ar/R constriction. We can find this tendency characterized by a high energetic barrier at the ar/R constriction conjugated with smaller increases of the other energetic barriers for cholecalciferol and calcitriol (Figure 3B).

## 4. Discussion

Recently, we highlighted the possible functional interaction existing between corticosteroids and AQPs through molecular dynamics approaches (28, 29). To our best knowledge, this was the first time evidence was brought in favor of such a direct modulation of AQP2 water permeability by a corticosteroid. From these initial works led on dexamethasone and cortisol and based on the structural similarity existing between GR, MR, the consensus cholesterol motif (CCM), and the extra-cellular vestibules of AQPs, we proposed an AQPs Corticosteroids Binding Site (ACBS) (29). The aim of the present study was to further investigate the relevance of the ACBS by confronting it to a larger sample of the steroid family through the same *in silico* methodology.

### 4.1. Additional evidence in favor of physiological relevance for the AQPs corticosteroids binding site

First of all, we observed a wide diversity in terms of affinity for the AQP2 extra-cellular surface between the steroids tested (Figure 1). This is in good accordance with the physiological regulation of AQPs by steroids as one would expect AQPs to be regulated differently depending on their type and hence on their function and tissular localization. Regarding AQP2, while cortisone, androsterone, oestradiol and cortisol have a very low affinity for its ACBS (median *K*_D_ of 590 nM, 544 nM, 562 nM, and 498 nM respectively), methylprednisolone, solu-medrol, and progesterone have a higher affinity (median *K*_D_ of 291 nM, 267 nM, and 289 nM respectively) and finally dexamethasone, calcitriol, and cholecalfciferol displayed the strongest affinity for AQP2 ACBS or the smallest *K*_D_ (median *K*_D_ of 193 nM, 114 nM, and 101 nM respectively). Moreover, all these *K*_D_ fall into the range of physiologically relevant drug–receptor interaction (45). The differences with our previous work mean *K*_D_ for dexamethasone [317.7 nM (28)] and cortisol [239.17 nM (29)] come from the methodology. In fact, we previously used portions of trajectories where the steroid was continuously bonded to the ar/R arginine to calculate *K*_D_ while in the present work, all the trajectory was taken into account to better discriminate the tested molecules. Finally, if we compare the median *K*_D_ for the two drugs with the best affinity for ACBS to the literature, similar values are found: Dexamethasone has a median *K*_D_ of 193 nM, and experimental *K*_D_ for glucocorticoid membrane receptors ligands was estimated at 180 nM (46); vitamin D3 has a *K*_D_ for its receptor equal to 32 nM (47) which is close to our estimated median *K*_D_ of 101 nM or 114 nM.

Second, this diversity was also observed in the functional impact of the interaction upon AQP2 water permeability (Figure 2). The traditionally used *pf* falls into the range of experimentally obtained AQP2 water permeabilities (3.3 ± 0.2 × 10^−14^ cm^3^ s^−1^ (12) compared to ~2 × 10^−14^ cm^3^ s^-1^, Figure 2). However, in a previous work, we noticed that the methodology used to derive *pf* from molecular dynamics simulations could be biased by the thermal agitation of water molecules (48). Thereafter we used another indicator for AQP2 water permeability consisting of the counts of water molecules crossing the whole trans-membrane pore section of the channel. With this alternative indicator, all tested steroids significantly reduced the permeability of AQP2. Moreover, the molecules were segregated between groups of different orders of magnitude of intensity of the impact. Coherently with a physiological modulation of AQPs by steroids, these groups correlated with the different steroid families represented: corticosteroids and androgens impacted the water permeability the less, followed by progestogens, estrogens, and finally vitamin D3 (Figure 2).

### 4.2. Physiological relevance for inner ear pathophysiologies: AQP2 is a good therapeutic target

AQP2 has been shown to be located in several inner ear tissues bordering the endolymph such as Reissner’s membrane, the organ of Corti, inner and outer sulcus cells, and the spiral limbus within the cochlea (49); or in the luminal epithelium of the endolymphatic sac (17, 18, 50). Initially discovered in the kidney and associated with the regulation of water re-absorption in the distal collecting duct (51–53), its localization in ES epithelium coincides with the hypothesis suggesting ES as the endolymph resorption site (54–58). In good agreement with similar roles of AQP2 in the kidney and the inner ear, similarities are found between the collecting duct and the ES epithelium. Both are composed of two epithelial cell types (59–63) and express many other ion transporters and channels (64–70). In the kidneys, AQP2 density at the plasma membrane of collecting duct principal cells is regulated through the action of the arginine vasopressin hormone (AVP, also known as antidiuretic hormone). Under binding to the vasopressin receptor (V2), the hormone induces the translocation of AQP2 from internal vesicles toward the plasma membrane (52, 71). Several yet published observations point toward a similar regulation of ES water permeability by AVP through the translocation and transcript level regulation of AQP2. Indeed, the V2 receptor as well as proteins known to mediate the translocation of intracytoplasmic vesicles into the plasma membrane are expressed in the ES (15, 18, 50, 72). Moreover, specific binding of AVP to the ES epithelium has been demonstrated by autoradiography (72). Finally, through AVP and protein kinase antagonists, this translocation of AQP2 has been shown in human ES (50). However, contrarily to the regulation taking place in the kidney, plasma AVP increase and subsequent V2R-cAMP-PKA-AQP2 activation induces endosomal trapping of AQP2 in the ES (50). This endosomal trapping of AQP2 is susceptible to decreasing ES luminal epithelium water permeability. As a consequence, this would negatively interfere with the re-absorption of endolymph AQP2 insures (in part) when needed, which could ultimately lead to endolymphatic hydrops (50). In Menière’s disease (MD), EH is usually observed (1), so much so that it is considered as a biomarker of the pathology (4, 5). Interestingly, in MD patients, elevated concentrations of AVP are found (68, 73). Coupled with the significant up-regulation of V2R and AQP2 genes in MD patients (50, 74, 75), these data point to AQP2 as a very interesting therapeutic target for inner ear pathophysiologies. However, in other studies, no significant increase in AVP plasma level was found for MD patients (76–78) making the role of AVP in MD and other inner ear endolymph fluxes regulation controversial.

### 4.3. Physiological relevance for inner ear pathophysiologies: functional hypothesis

**Figure 4:**
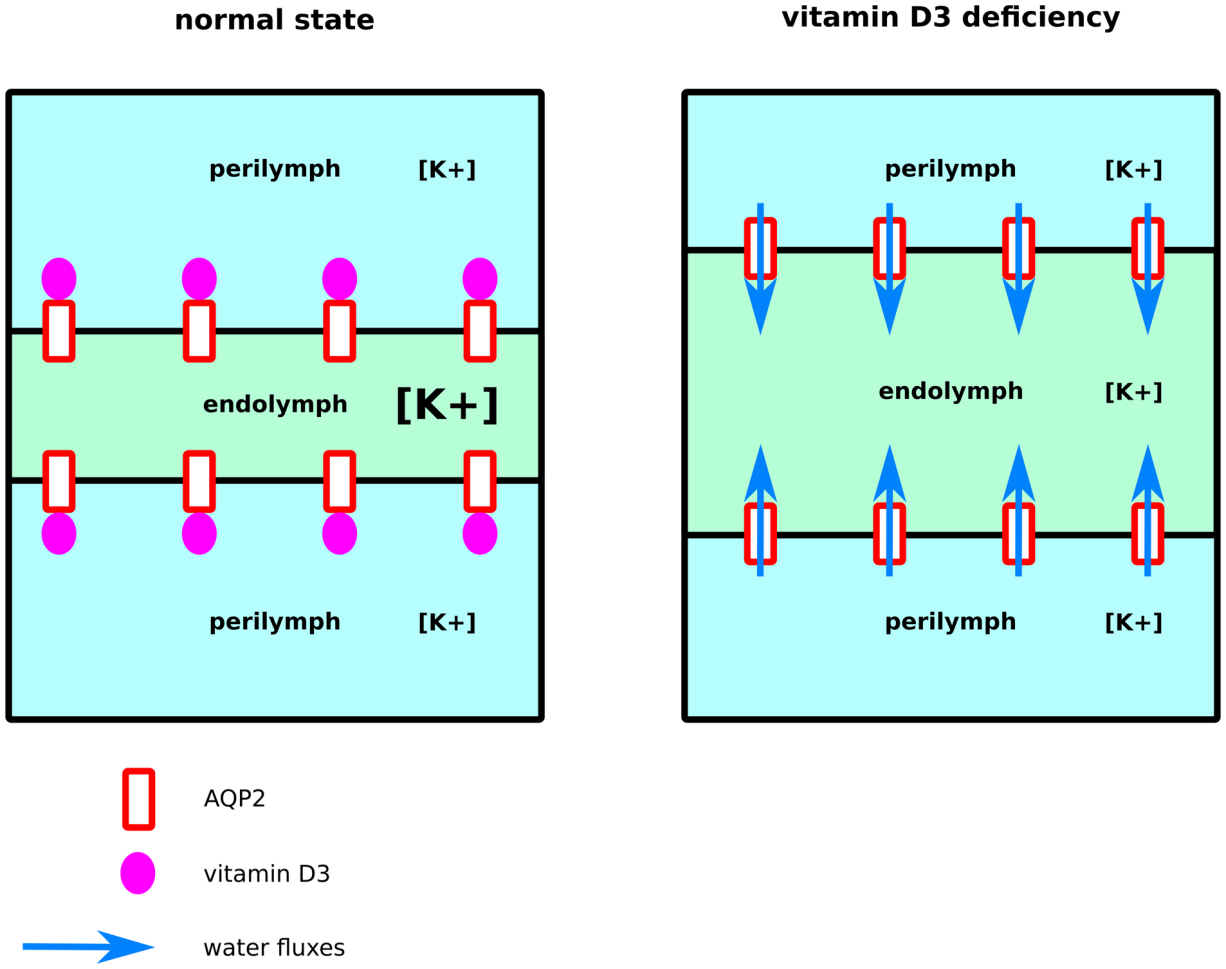
Schematic illustration of our functional hypothesis for vitamin D3 – deficiency mediated endolymphatic hydrops. In normal conditions, vitamin D3 inhibits AQP2-mediated trans-cellular water flux hence maintaining cochlear canal (in green) volume. In the case of a vitamin D3 deficiency, however, water fluxes follow the osmotic gradient and flow from the surrounding perilymphatic compartments (in blue) toward the cochlear canal leading to endolymphatic hydrops.

Endolymph is a unique extra-cellular hyperosmotic fluid with unusual characteristics such as a very high potassium concentration and low tightly controlled calcium concentration (9, 79). Because hair cells have their apical membrane protruding within one of the endolymphatic compartments called the cochlear canal, endolymph volume, and K^+^ and Ca^2+^ concentrations must be kept constant for the endocochlear potential to be generated and hence normal auditory functions to be maintained (9, 79–81). Perilymph is on the other hand isotonic and possesses ionic concentrations similar to what is found in the cytosol of cells (79). To maintain these ionic concentrations, active transport is made by cells of tissues surrounding the cochlear canal (58, 79). According to direct measurements of the dispersal of markers in endolymph, these ion fluxes are not accompanied by water fluxes, and in normal conditions, endolymph is maintained without significant changes in volume (58). Thereafter, according to this hypothesis, water permeability of the inner tissues surrounding the cochlear canal should stay very low. Moreover, because of the calcium level regulation taking place in these same inner ear tissues, vitamin D3 is also expected to be concentrated. Indeed, vitamin D3-carrier protein complexes such as calbindin 28 kDa as well as endocytotic receptors involved in these vitamin-D-carrier protein complexes intakes, such as cubilin and megalin, are found in many cochlear cells (82–86). To summarize, much evidence indicates that both AQP2 and vitamin-D3 co-localize into the inner ear cells surrounding the cochlear endolymphatic compartment. In the present study, our results suggest vitamin-D3 active and inactive forms as strong inhibitors of AQP2 water channeling activity. Indeed, both cholecalciferol and calcitriol directly interacted with the predicted AQP2 ACBS; presented the strongest inhibition of AQP2 water permeability through both *pf* (for cholecalciferol only) and water counts (Figure 2), and presented the highest affinity for the ACBS (Figure 1) which was very close to the affinity for its known receptor. This was clearly highlighted by our new water fluxes inhibitory potential indicator (*WIP*, Figure 2). The second most significant inhibition of AQP2 function was observed with oestradiol. Interestingly, the fluctuation of these two steroid hormones has been correlated to MD symptoms (87–91). Coherently, in both cases, the steroid hormone deficiency was associated with hearing impairment (92–95) and vertigo (96, 97). Moreover, a recent study reported no significant difference in the allelic frequency of estrogen receptor ERα polymorphisms between patients with MD and controls (98) suggesting these effects being mediated through another pathway. In another recent study, vitamin D deficiency in the elderly but not total calcium concentration was significantly associated with hearing losses (95) thereafter re-enforcing the likeliness of a direct effect of this hormone on inner ear homeostasis. Hence, we formulate the hypothesis that steroids and in particular vitamin D3 and/or oestradiol have a physiologically relevant inhibitory action upon AQP2 water channeling and that this regulation is directly linked to endolymphatic hydrops, MD symptoms, and other related inner ear pathophysiologies (Figure 4): in normal conditions, endolymphatic volume is stable (58) and because the cochlear canal endolymph is hyperosmotic compared to perilymph (9, 58, 79), membrane water permeability of the surrounding cells must be very low. In these cells AQP2 is expressed (49) and many ionic canals and transporters are needed to maintain the endocochlear membrane potential (79). Because of the Ca^2+^ regulation taking place in these cells, vitamin D3 is also present and actively concentrated by dedicated endocytosis (86). On top of its role in calcium cycling, vitamin D3 directly interacts with AQP2 ACBS (Figure 1) which inhibits water fluxes (Figure 2) through the modification of the conducting pore electrostatic profile and diameter (Figure 3) (28, 29). This inhibition of AQP2 water permeability hinders trans-cellular water fluxes between perilymph and endolymph. In pathological conditions, however, when the patient displays vitamin D and/or oestradiol deficiency, calcium cycling is impaired and water fluxes between perilymph and endolymph can occur through the AQP2-mediated trans-cellular route. Following the osmotic gradient, water would flow from the perilymph toward the endolymph hence resulting in endolymphatic hydrops. In turn, both volume and ionic concentrations of the endolymphatic compartment would have been severely impaired leading to hearing loss and vertigo symptoms (87–90, 92–94, 96, 97).

### 4.4. Physiological relevance for inner ear pathophysiologies: limits and remaining interrogations

In the current paper, we formulated a functional hypothesis relevant to inner ear pathophysiology based on molecular dynamics simulation experiments. However many more investigations are needed to validate this hypothesis. Firstly, the modulation of AQP2 by steroids needs to be tested through other approaches *in vitro* and *in vivo*. Secondly, even though AQP2 and vitamin D3 are very likely to co-localize in tissues bordering the endolymph of the cochlear canal, other AQPs (AQP1, AQP3, AQP4, AQP5, AQP6, AQP7, and AQP9) are also expressed in these same tissues (99, 100). Moreover, knowing precisely how much each subtype of AQP contributes to the global water exchanges between the two compartments is still very challenging. Furthermore, additional information about the exact cellular localization of each AQP and of vitamin D3 will eventually be needed to corroborate our hypothesis. On top of tissular and cellular localization, the way vitamin D3 and other steroids could modulate other AQPs’ water permeability is still unknown. Some of them could display a ubiquitous inhibition upon AQP function while others could impact different subtypes in an opposite way. We should also remember that recent data indicate that steroids could directly modulate the function of other ionic channels as well, such as members of the Transient Receptor Potential (TRP) channels (101, 102). Interestingly, these mechanosensitive calcium channels have been shown to form functional complexes with AQPs (103) and are known to co-localize with AQP2 in human endolymphatic sacs (18). Thirdly, other factors could interfere with AQP function in physiological conditions, such as ion concentrations (48, 104) and pH (105, 106). In the inner ear, the endolymphatic compartment is characterized by a very high potassium ions concentration tightly maintained at nearly 150 mM from which originates the endolymphatic potential (9, 79–81). Additionally, the endolymphatic pH is kept at 7.4 (107). In the present study, the AQP2 tetramer was inserted into a POPC bilayer, solubilized with 150 mM of KCl ions and the histidine residues (protein pH sensors) were simulated with an unprotonated imidazole side-chain. Our atomic systems hence constitute non-exhaustive models of inner ear physiological conditions with some limits such as the choice of potassium as the only representative cation; the bilayer lipidic composition; and the absence of other interacting partners such as calcium or other proteins. These choices stem from the necessary trade-off between the accuracy of the systems simulated and the associated computational costs.

Finally, we should keep in mind the strong anti-inflammatory effect of steroids, which remains to this day the main molecular mechanism put forward to explain the efficiency of MD corticosteroid treatments. The significant role of inflammation in the disease is supported by many evidence (108, 109). However, contrary to the non-genomic effects described in the present study, this inflammation involves the modulation of gene expression (110). It is however worth noting that a growing body of evidence is questioning the efficacy of dexamethasone and other corticosteroids effect in MD treatment and EH resorption (27, 111, 112). Even more puzzling is the fact that these studies represent the largest high-quality trials of a nondestructive drug treatment for patients with MD and that the protocols were designed in order to maximize the efficiency of dexamethasone delivery at the site of EH formation (111). To explain these discrepancies, the authors put forward two hypotheses: either the highly associated placebo effect masked the effect of corticosteroid treatment, or the recording time frame was not appropriate (111). Our hypothesis could also bring an explanation regarding this failure to alleviate EH with dexamethasone: if the corticosteroid indeed significantly modulates AQP water permeability, then the precise tissular application site, concentration of the molecule, moment of application relatively to EH formation and the nature of the corticosteroid itself could all significantly impact treatment efficiency. Following this idea, high and sustained delivery of dexamethasone in the middle ear could also strongly inhibit AQP2-mediated water fluxes in both the cochlea and the ES. If the EH was already established within the treated ears, then high dexamethasone concentrations could have prevented EH resorption because of lymph fluxes inhibition. This hypothesis is supported by a very recent study that followed EH evolution through MRI after intratympanic injection of dexamethasone in patients with probable or definite MD (112). The authors reported no effect of dexamethasone on EH resorption (112). It is however still a matter of debate whether EH is at the basis of MD symptoms or if it can only be considered as a side effect of the disease.

### 4.5. Conclusive remarks

Altogether these data support the physiological relevance of AQP2–steroids interactions as a non-genomic regulation of lymphatic fluxes between perylymphatic and endolymphatic compartments at the origin of EH. Further investigations and trans-disciplinary approaches are needed to confirm this functional hypothesis. Among the ten molecules tested, vitamin D3 displayed the most significant functional inhibition of AQP2 water fluxes. During the previous years, more and more data linked vitamin D3 deficiency with hearing losses (93–95), vertigo (88, 97), and MD (87, 91, 113). Most of them however are cross-sectional studies and the demonstration of a direct causal relationship between vitamin D3 deficiency and EH formation is still missing. However, we hope that by highlighting the plausible direct regulation of water and ions homeostasis by steroid interaction with transmembrane channels, a re-investigation of inner ear physiology will be conducted to eventually bring significant treatment improvements.

## Data availability statement

The raw data supporting the conclusions of this article will be made available by the authors, without undue reservation.

## Author contributions

RM: Conceptualization, Data curation, Formal analysis, Investigation, Methodology, Software, Writing – original draft. SR: Funding acquisition, Methodology, Project administration, Resources, Supervision, Writing – original draft. VM: Funding acquisition, Project administration, Resources, Supervision, Validation, Writing – review & editing. DA: Conceptualization, Investigation, Methodology, Supervision, Validation, Writing – review & editing.
